# Three-dimensional changes in left atrial volumes and ejection fraction during dobutamine stress Cardiovascular Magnetic Resonance

**DOI:** 10.1186/1532-429X-11-S1-P176

**Published:** 2009-01-28

**Authors:** Vinodh Jeevanantham, William Ntim, Craig Hamilton, Tim Morgan, John Hoyle, Gregory Hundley

**Affiliations:** Wake Forest University, Winston Salem, NC USA

**Keywords:** Atrial Fibrillation, Left Atrial, Dobutamine Stress, Left Atrial Volume, Cardiac Risk Factor

## Introduction

Left ventricular performance is routinely measured during dobutamine cardiovascular magnetic resonance (DCMR) and the stress induced left ventricular changes have been well described. Left atrial (LA) size and volume are associated with increased risk of developing atrial fibrillation, stroke and congestive heart failure; however changes in LA volume and LA ejection fraction (LAEF) during DCMR have not been described.

## Purpose

To determine LA volumes and LAEF in patients undergoing DCMR.

## Methods

We enrolled 128 consecutive participants referred for DCMR. Using white blood CINE steady-state free precession imaging, a 3 dimensional LA model (determined from a multi-slice, multi-phase stack of LA short axis views) was obtained to compute LA volumes and LAEF throughout dobutamine stress. Baseline clinical cardiac risk factors such as diabetes mellitus, hypertension, coronary artery disease (CAD), atrial fibrillation, age and gender were collected. Dobutamine stress protocol involved infusion of 7.5 μg/kg/min for low dose stage and 20 μg/kg/min ± atropine for peak dose stress stage. LA maximal volume (LAV max) was defined as maximal LA volume before the opening of mitral valves. LA minimal volume (LAV min) was defined as minimal LA volume at closure of mitral valve. LAEF was defined as (LAV max-LAV min)/LAV max. All volumes were indexed to body surface area. One-way Analysis of variance (ANOVA) was used to detect the differences in LA volumes and LAEF across stages of DCMR. Linear regression analysis was used to find the independent predictors of LAEF and LA volumes.

## Results

The age of our participants averaged 69 ± 8 years; 51% were men. Twenty seven percent of the participants had CAD, 33% had diabetes, 88% had hypertension, and 7% had atrial fibrillation. Mean LA volumes and LAEF changed at each stage of DCMR (Table 1). Cardiac risk factors influencing LA volumes and LAEF included: CAD (p = 0.04), male gender (p = 0.04), and atrial fibrillation (p = 0.02). Also, increasing age was inversely correlated with LAEF [baseline (p = 0.01), low dose (p = 0.004) and peak dose (p = 0.19)]; and positively correlated with LA volumes; LAV Min [baseline (p = 0.01), low dose (p = 0.02) and peak dose (p = 0.01)]. Regression analysis revealed that the significant predictors for baseline LAEF were atrial fibrillation (p 0.01), and CAD (p 0.05); for low dose LAEF were increasing age (p 0.02), and atrial fibrillation (p 0.003); and for peak dose LAFE was age (p = 0.01). After adjusting for all confounding variables, increasing age was the most significant predictor for LA volumes across all stages of stress [baseline (p 0.05), low dose (p 0.03) and peak dose (p 0.02)].

**Table 1:** Mean differences in left atrial volumes and ejection fraction during dobutamine CMRI

	Baseline (N = 128)	Low dose (N = 126)	Peak dose (N = 105)	P value
LAV max	33.1 ± 8.7	31.5 ± 9.7	26.3 ± 8.1	< 0.001
LAV min	19.9 ± 5.9	17.8 ± 6.3	16.8 ± 6.2	< 0.001
LA EF	40.1 ± 6.9	43.6 ± 7.3	36.6 ± 10.2	< 0.001

## Conclusion

Left atrial volumes decrease during progressive stress levels of DCMR. Presence of underlying CAD or atrial fibrillation significantly decreased LA volumes and left atrial ejection fraction. Increasing age was associated with increased LA volumes but decreased LA ejection fraction. Further studies to understand the prognostic value of these changes are needed.

